# Comparative evaluation of ultrasound-guided versus operative drainage for pediatric appendicular abscess: Effectiveness, safety and clinical outcomes

**DOI:** 10.6026/973206300214974

**Published:** 2025-12-15

**Authors:** K.M Shaiful Islam, Nazmus Sakib Ferdous, Syeda Tamanna Tanjil, Md Rassell, Zannatul Ferdous Peu, Ashraf Ul Huq

**Affiliations:** 1 Department of Paediatric Surgery, Bangladesh Medical University, Dhaka, Bangladesh; 2 Department of Paediatric Surgery, Dhaka Medical College Hospital, Dhaka, Bangladesh; 3 Department name missing, Bangladesh Institute of Research and Rehabilitation in Diabetes, Endocrine and Metabolic Disorders, Shahbag, Dhaka, Bangladesh; 4 Department of Surgical Oncology, Bangladesh Medical University, Dhaka, Bangladesh

**Keywords:** Appendicular abscess, ultrasound-guided drainage, operative drainage, pediatric surgery, post-operative complications

## Abstract

The optimal management of appendicular abscess in children remains a challenge, with ongoing debate over the most effective treatment approach. Therefore, it is of interest to compare ultrasound-guided drainage (UD) and operative drainage (OD) for the treatment of appendicular abscess in children. Sixty patients were analyzed, with 30 undergoing OD and 30 undergoing UD. The UD group had significantly lower pain scores, body temperature and white blood cell counts, along with a shorter hospital stay compared with the OD group. Post-procedural complications were more frequent in the OD group. Thus, we show that ultrasound-guided drainage is a safe and superior alternative to operative drainage for appendicular abscess in children.

## Background:

Acute appendicitis is one of the most common surgical diseases in the pediatric population and presents with a wide spectrum of symptoms and pathologies [1, 2]. Delayed diagnosis can lead to serious complications such as perforation, abscess formation, peritonitis, wound infection, sepsis, infertility, adhesions, bowel obstruction and death [3]. An appendicular abscess represents a form of complicated appendicitis in which a localized abscess develops around the appendix [4, 5]. Management of appendicular abscess remains controversial, particularly regarding immediate surgery versus conservative treatment followed by interval appendectomy [5, 6]. At present, interval appendectomy is usually performed only in patients with recurrent symptoms [7, 8]. In children, typical clinical signs of acute appendicitis are not always evident and physical examination findings may be misleading [9]. Such misinterpretation often results in delayed diagnosis and progression to perforation or gangrene with abscess formation [10]. Appendicular abscess can be treated by operative drainage (OD), which is associated with several risks. These include hemorrhage, wound infection and delayed wound healing, injury to surrounding structures and anesthetic hazards. Open procedures also result in longer hospital stay and increased psychosocial and financial burden [11]. With advances in imaging technology, ultrasound- guided percutaneous drainage (UD) has emerged as an effective alternative to open drainage. In experienced hands, UD is minimally invasive and cost effective for the treatment of appendicular abscess [12]. Previous studies have reported sensitivity greater than 85% and specificity greater than 90% for this technique. Many patients treated with percutaneous drainage complete management with antibiotics alone, without requiring further procedures [13, 14]. Therefore, ultrasound-guided drainage may provide a more effective and less invasive management option for appendicular abscess in the pediatric population. Therefore, it is of interest to assess the outcomes of ultrasound-guided drainage and operative drainage in children with appendicular abscess.

## Materials and Methods:

This was a prospective comparative interventional type of study. Simple random sampling technique was chosen by performing lottery procedure. A total of 60 (30 in each group) patients were included in this study. The study took place in the department of Pediatric Surgery, Dhaka Medical College Hospital (DMCH), Dhaka, Bangladesh from January 2017 to December 2018.

## Inclusion criteria:

[1] All Children with appendicular abscess from 02 years to 12 years of age.

[2] Given informed written consent.

## Exclusion criteria:

[1] Patients with congenital disability or major co-morbid disorder or disease.

[2] Patients with systemic sepsis or ICU admission.

[3] Refusal of patients or patients' parents or the researcher at any stage.

[4] Appendicular abscess in multiple site of peritoneal cavity.

## Study Procedure:

Patients were prepared after admission and kept nil per oral. All patients were examined by at least two different examiners. Investigations were performed according to the standard protocol. All patients included in the study were classified as American Society of Anesthesiologists (ASA) grade I or II. For operative drainage, careful physical examination was performed in the operating room under anesthesia. Patients were encouraged to evacuate the bladder before the procedure.

## Operative drainage (Extraperitoneal Drainage):

The patient was placed in the supine position on the operating table. Parenteral prophylactic antibiotics were administered at the time of induction according to protocol. General anesthesia was used. Aseptic preparation from the mid-chest to mid-thigh was performed using povidone iodine. The operative field was draped in the usual sterile manner. In extra peritoneal drainage, a small muscle-cutting iliac incision was made lateral to the summit of the mass. All muscle layers were divided along the line of the skin incision. The peritoneum was often thickened and edematous. It was incised carefully, as it might be adherent to the underlying mass. The medial leaf of the peritoneum was retracted forward and a gauze pack was placed beneath it. The mass was gently explored with a finger directed laterally and backward. The abscess cavity was usually identified without difficulty. A variable amount of malodorous pus was evacuated. Pus samples were collected and sent for culture and sensitivity testing. Adhesions forming loculi within the cavity were gently broken with the finger. A soft drain tube was inserted into the cavity. The wound was loosely closed around the drain. In the OD group, drainage was performed gently and the approximate amount and color of pus were noted.

## Ultrasound-guided drainage:

The drainage technique was selected by the interventionalist. The approach depended on abscess location, interposed organs and operator expertise. The anterior abdominal transperitoneal approach was most commonly used. Route planning involved careful consideration of the cutaneous entry site, angle and depth. After diagnostic scanning using the TH-80 Ultrasound Diagnostic System, drainage routes were planned. A straight line from the skin entry site to the abscess wall was ensured. Following sterile skin preparation, the procedure was performed under local anesthesia. A puncture needle with a stylet (20 cm, 18G = 1.2 cm; Angiotech, Stenløse, Denmark) was introduced into the abscess cavity under continuous ultrasound guidance. Pus was aspirated using a syringe and sent for immediate Gram stain and culture. In the UD group, each patient underwent diagnostic scanning for adhesions and other abnormal collections or masses. In female patients, both ovaries were also examined.

## Post-procedural management and follow-up:

All patients received intravenous ampicillin (100 mg/kg/day), amikacin (15 mg/kg/day) and metronidazole (1.5 mg/kg/dose) for the first five postoperative days. From the sixth postoperative day onward, oral cefixime (10 mg/kg/day) was administered. For analgesia, injectable pethidine (1.5 mg/kg/dose) was given on the first postoperative day. Per-rectal diclofenac sodium (1.5 mg/kg/dose) was continued until pain subsided. Post-procedural follow-up included assessment of pulse, temperature, wound dressing, pain and bowel sounds. Discharge criteria included absence of wound complications, fever and procedural pain. A total of five follow-up visits were conducted. These occurred on postoperative day (POD) 0, POD 3, POD 5, POD 6 and POD 30 after discharge.

## Statistical analysis of data:

Statistical analysis was performed using Statistical Package for the Social Sciences (SPSS) version 22.0. Descriptive analysis was conducted for clinical features. Quantitative variables were expressed as mean ± standard deviation. Qualitative variables were presented as numbers and percentages. The chi-square test and unpaired t-test were used as appropriate. All tests were two-sided. A p- value < 0.05 was considered statistically significant.

## Ethical clearance:

Ethical approval was obtained from the Ethical Review Committee of Dhaka Medical College. Permission was also taken from the concerned departments. Informed written consent was obtained from all parents or legal guardians. The purpose of the study was clearly explained to them.

## Results:

Table 1 (see PDF) shows the demographic profile of the study population. The mean age of children underwent UD group was 9.16 ± 2.05 years, while that of the OD group was 8.83 ± 2.21 years, with no statistically significant difference between the groups (P = 0. 548). The age range was 5-12 years in the UD group and 4-12 years in the OD group. In terms of sex distribution, males predominated in both groups-21 (70.0%) in the UD group and 17 (56.7%) in the OD group-while females accounted for 9 (30.0%) and 13 (43.3%), respectively. The difference in sex distribution between the two groups was not statistically significant (P = 0.284). Table 2 (see PDF) illustrates the pain status before and after intervention in both groups. The mean pre-procedural pain score was comparable between the UD group (9.53 ± 0.86) and the OD group (9.40 ± 0.93), with no significant difference (P = 0.567). However, by the 3rd postoperative day (POD), the UD group showed a significantly lower pain score (6.93 ± 1.46) compared to the OD group (8.60 ± 1.30; P < 0.001). On the 6th POD, the mean pain score remained significantly lower in the UD group (3.20 ± 1.71) than in the OD group (4.20 ± 1.76; P = 0.030). Table 3 (see PDF) summarizes the changes in body temperature before and after the procedures. The mean pre- procedural temperature was comparable between the UD group (103.46 ± 0.59°F) and the operative drainage (OD) group (103.34 ± 0.62°F), with no significant difference (P = 0.460). By the 3rd postoperative day (POD), both groups showed a marked reduction, with mean temperatures of 99.45 ± 1.22°F in the UD group and 99.71 ± 0.98°F in the OD group; the difference was not statistically significant (P = 0.366). On the 6th POD, however, the UD group demonstrated a significantly lower mean temperature (98.67 ± 0.58°F) compared with the OD group (98.97 ± 0.54°F; P = 0.043). Table 4 (see PDF) presents the total leukocyte count (TLC) before and after intervention in both groups. The mean pre-procedural TLC was similar between the UD group (15,483 ± 1,816) and the OD group (15,403 ± 1,974), with no significant difference (P = 0.870). By the 5th POD, the UD group showed a significantly lower mean TLC (9,452 ± 895) compared to the OD group (10,220 ± 1,400; P = 0.014), indicating a faster resolution of infection in patients undergoing ultrasound-guided drainage. Table 5 (see PDF) compared the neutrophil count before and after procedure in UD and OD group. Here pre procedural/operative neutrophil count was similar in both groups [UD= 80.38 ± 4.20, OD= 80.25 ± 4.31, p value= 0.906 (95%CI)]. At 5th PPD/POD, neutrophil count in UD group (70.87 ± 4.69) was significant [p-value =0.003 (95%CI)] than OD (74.66 ± 4.85). Table 6 (see PDF) shows the duration of postoperative hospital stay in both groups. Children treated with ultrasound-guided drainage (UD) had a significantly shorter mean hospital stay (5.17 ± 0.38 days) compared to those who underwent operative drainage(OD), whose mean stay was 5.93 ± 2.02 days (P = 0.045). Table 7 (see PDF) outlines the postoperative wound complications in both groups. No wound-related complications were observed in the UD group, whereas 6 patients (20%) in the OD group developed wound complications and this difference was statistically significant (P = 0.023). Among the OD group, disturbance of wound healing was seen in 1 patient (16.7%), moderate wound infection in 3 patients (50.0%) and severe wound infection in 2 patients (33.3%), though these differences did not reach statistical significance (P > 0.05). In contrast, satisfactory wound healing was significantly more frequent (24 patients, 80.0%; P < 0.001). In Table 8 (see PDF), post-operative wound status in operative drainage group are shown. In OD group (n= 30), 6 patients (20%) had wound complications. Of them one patient (16.67%) had superficial wound infection. three patients (50%) had incomplete wound dehiscence and another two patients (33.33%) had complete wound dehiscence. Other 24 patients (80%) had no wound complications. Table 9 (see PDF) summarizes the bacteriological profile of pus samples collected from both groups. In the UD group, the majority of cultures showed no bacterial growth (26 cases; 86.67%), while in the operative drainage (OD) group, 23 cases (76.67%) had sterile cultures. Among culture-positive cases, Escherichia coli was the most frequently isolated organism-3 cases (75%) in the UD group and 5 cases (71.43%) in the OD group. Klebsiella species were also found in 1 case (25%) in each group. Additionally, Pseudomonas aeruginosa was identified in 1 case (14.28%) from the OD group, but not in the UD group.

## Discussion:

This prospective study evaluated postoperative outcomes between UD and OD in 60 pediatric patients aged 2-12 years with appendicular abscess. Patients were randomly assigned by lottery to UD (n=30) or OD (n=30). Children younger than 2 years were excluded, consistent with literature reporting rare appendicitis complications in this age group [15, 16]. Demographic characteristics were comparable between the two groups. The mean age was 9.16 ±2.05 years in the UD group and 8.83±2.21 years in the OD group. Male predominance was observed in both groups, though it was higher in the UD group (70%) than in the OD group (56.7%). Patients treated with UD reported significantly lower pain scores on the Wong-Baker FACES scale. This difference was evident on the 3rd postoperative day (6.93±1.46 vs. 8.60±1.30; p<0.001). It persisted on the 6th postoperative day (3.20±1.71 vs. 4.20±1.76; p=0.030). Similar trends have been reported in previous studies, although lower absolute pain scores were noted with enhanced analgesic protocols [17]. Admission body temperatures were similar between groups. UD patients demonstrated faster normalization of temperature by the 6th postoperative day (p=0.043). This finding is consistent with earlier reports [1]. Leukocyte and neutrophil counts declined more rapidly in the UD group. By the 5th postoperative day, WBC counts were significantly lower in UD patients (9452±895 vs. 10220±1400; p=0.014). These results align with prior observations [4, 8]. Hospital stay was significantly shorter in the UD group. The mean duration was 5.17± 0.38 days for UD patients compared with 5.93±2.02 days for OD patients (p=0.045). Longer hospitalization in the OD group was associated with postoperative wound complications. Wound infection occurred in 20% of OD patients and none in the UD group, as assessed by ASEPSIS scoring. Escherichia coli were the most commonly isolated organism in both groups. It was identified in 75% of UD and 71.43% of OD culture-positive cases. No mortality or 30-day postoperative complications were observed in either group. Overall, UD demonstrated better pain control, faster recovery and fewer wound complications. These findings support UD as the preferred approach where appropriate expertise and resources are available. Limited access to interventional radiology and traditional surgical training may still restrict widespread adoption.

## Limitations:

[1] Due to confined study period, long time follow up could not be performed.

[2] Sample sizes of 60 cases analyzed in this study were not adequate to calculate exact figure.

[3] Ultrasound- guided drainage and operative drainage was not performed by the same surgeon or intervention radiologist.

[4] Failure to attend on exact day of follow up, due to problem of transportation, remote areas from the center of study, standard follow-up schedule could not be maintained in all cases.

[5] Single center analysis was performed.

## Recommendation:

As ultrasound- guided drainage is beneficial for appendicular abscess, it should be the first choice of treatment option for drainage of appendicular abscess in children.

## Conclusion:

Ultrasound- guided drainage is safe and can provide less post-operative morbidity in experienced hands, than operative drainage in case of appendicular abscess in children. Most cases of appendicular abscess can be treated by ultrasound- guided drainage except for those who have contraindication. Ultrasound- guided drainage is a useful method for reducing hospital stay, good pain management, temperature control and avoiding complications.
